# Necessity and Reconstruction Methods of Splenic Vein After Resection of the Portomesenteric Junction During Resections for Pancreatic Cancer

**DOI:** 10.3390/curroncol32060316

**Published:** 2025-05-30

**Authors:** Moath Alarabiyat, Nikolaos Chatzizacharias

**Affiliations:** 1 Department of HPB and Liver Transplant Surgery, Queen Elizabeth Hospital Birmingham, Birmingham B15 2GW, UK; moath.alarabiyat@nhs.net; 2 Institute of Clinical Sciences, College of Medical and Dental Sciences, University of Birmingham, Birmingham B15 2TT, UK

**Keywords:** pancreatic cancer, splenic vein ligation, splenic vein reconstruction, sinistral portal hypertension, venous resection, pancreatic resection

## Abstract

Pancreatic cancer involving the porto-mesenteric junction (PMJ) represents a challenge to pancreatic surgeons. Restoring mesenteric venous drainage is an essential component of vascular reconstruction after tumour resection. In contrast, management of the splenic venous drainage can involve the ligation or reconstruction of the splenic vein (SV). Evidence suggests that splenic vein ligation (SVL) is commonly associated with sinistral portal hypertension (SPH), especially if multiple venous tributaries were divided to facilitate resection. Although the association between SVL and SPH is well documented, the risk of symptomatic SPH is not widely reported, presumably due to the low incidence and poor survival of pancreatic cancer patients. Splenic vein reconstruction (SVR) has been proposed to decrease the risk of SPH but is fraught with technical complexity and increased morbidity. Moreover, SVR does not guarantee the prevention of SPH, as patency rates vary and associated hemodynamic changes are unpredictable. Patient selection and the surgical expertise available can guide SV intraoperative management, taking into consideration the risks and benefits associated with each approach. A comprehensive review of the current literature highlighting the incidence and clinical impact of SPH after the resection of pancreatic cancer involving the PMJ is presented.

## 1. Introduction

The anatomical delineation of splenic vein (SV) drainage is an essential component in the surgical planning of pancreatic cancer cases involving the porto-mesenteric venous junction (PMJ). This guides decisions regarding the suitability of splenic vein ligation (SVL) or reconstruction (SVR) and helps to predict the complexity of venous reconstruction if needed. Detailed knowledge of the splenic and mesenteric venous drainage system is readily available with new computed tomography (CT) scanners, which can provide two- and three- dimensional images [1].

The primary consideration in managing the SV is the risk of clinically significant or symptomatic sinistral portal hypertension (SPH). However, the existing literature presents conflicting evidence, with some studies reporting no increased risk of symptomatic SPH following SVL [2,3,4,5], while others advocate for reconstruction to mitigate short- and long-term complications [6,7,8]. After SVL, splenic blood flow is diverted through several routes, including the left gastric vein (LGV) via the short gastric veins; the inferior mesenteric vein (IMV); the middle colic and superior right colic veins (MCV, SRCV) via the arc of Barkow; the transverse colic marginal vein; and in rare cases, a naturally occurring splenorenal (SR) shunt [5,9,10,11,12]. Additionally, various unnamed collaterals can be identified in cases where the aforementioned alternatives are divided due to tumour involvement [2,13].

SVL is commonly favoured during pancreatic cancer resection due to the presence of collateral pathways that typically prevent significant venous congestion. While this approach avoids the complexity and potential morbidity of a complex venous reconstruction, SVR may be considered in selected cases to preserve venous outflow and reduce the risk of SPH. This review aims to critically evaluate the current evidence on the techniques and outcomes of SVL versus SVR, highlighting evidence where the occurrence and timing of symptomatic SPH have been reported in pancreatic cancer resection.

### What Is the Importance of SV Management in the Context of Contemporary Pancreatic Cancer Care?

Worldwide, pancreatic cancer ranks as the 12th most common cancer [14]. With advancements in cross-sectional imaging and cancer screening programs, along with increasing life expectancy, the incidence of pancreatic cancer is expected to rise. Surgery remains the only curative option and the evidence supporting this is well established in literature. While only around 20% of cases present at a resectable disease stage, the remaining patients will have evidence of local vascular involvement (borderline or locally advanced stage disease) or metastatic disease that is not amenable to standard surgical resections [15].

The advent of more effective systemic treatment options has allowed an increase in the number of patients with vascular involvement able to undergo surgical treatment. However, this is thought to be at the expense of more challenging procedures and vascular resection techniques that attain negative resection margins. Vein resections are now considered the standard of surgical care when tumour involvement is present. In cases of PMJ involvement, the management of the SV, ligation or reconstruction are important and should be decided upon by comparing the risks associated with a complex venous reconstruction against the risks associated with long-term symptomatic SPH.

Current evidence points to the fact that symptomatic SPH is an uncommon occurrence; this was demonstrated, for example, in a systematic review by Petrucciani et al. [16] and a large cohort study from Japan by Mizuno et al. [17], which we highlighted in our discussion. The main aim of this review is to present the published evidence on the clinical outcomes of SV management during pancreatic resectional surgery, such as the differences in the risk profile between SVR and SVL, and the incidence and consequences of SPH, including gastrointestinal bleeding and treatment options. This will assist surgical decision-making and an individualized approach to these cases.

## 2. Methodology

This is a narrative review of the existing literature on the consequences of either SVL or SVR in patients undergoing resection for pancreatic cancer. A literature search of the PubMed database until December 2024 was conducted to identify studies that discussed the outcomes of either SV ligation or reconstruction in pancreatic cancer patients. The terms used were as follows: “(Splenic vein) AND ((Pancreatic cancer) OR (Pancreatic ductal adenocarcinoma))”, “((Splenic vein ligation) OR (Splenic vein reconstruction)) AND ((Pancreatic cancer) OR (Pancreatic ductal adenocarcinoma))” and “((Sinistral portal hypertension) OR (Left sided portal hypertension)) AND ((Pancreatic cancer) OR (Pancreatic ductal adenocarcinoma))”.

Review articles, editorials, opinion statements and non-English language studies were excluded. Only reports that included pancreatic cancer cases involving SVL and/or SVR were included. When reports included patients with diagnoses other than pancreatic cancer, this was highlighted. The outcomes of interest in the included studies were the development of biochemical (low platelets), radiological (splenomegaly, varices) or clinical (bleeding) evidence of SPH. Other outcomes such as the time to the onset of symptomatic SPH, the requirement for intervention for symptomatic SPH, methods of SVR, and patency rates were also noted.

The data extracted from the reports were the year of publication, the number of patients who underwent either SVL or SVR, the factors on which SPH diagnosis was based on, incidence of SPH (asymptomatic or symptomatic) and median time to the onset of SPH symptoms. The results from 19 studies and 1 systematic review were noted. As this study was based on a review of the existing literature, no new data were generated and no statistical analysis was conducted.

### Definition of Sinistral Portal Hypertension

There has been no consensus agreement on what splenic pressure values or exact radiologic features constitute SPH. The diagnosis of SPH requires SV thrombosis or ligation accompanied by spleno-portal collaterals in the absence of clinical or radiologic features of liver cirrhosis. According to CT scan criteria, veins 5 mm or greater in diameter are considered enlarged [18]. Various parameters were used to assess the presence of SPH after pancreatic resection, such as the splenic volume, platelets count and variceal formation [2,4,5,8,19,20]. A variceal grade based on the number and diameter of the largest vessels has also been described in liver transplantation settings [21] and used to describe the varices developing after SVL in pancreaticoduodenectomy [22]. Radiologic indicators to predict the risk of gastrointestinal (GI) bleeding in SPH after pancreatic resection have not been identified but represent a potential area for future research.

## 3. Methods to Address SV Involvement

### 3.1. Splenic Vein Ligation

SVL in pancreatic cancer involving the PMJ is a complex decision influenced by several factors. Venous resection to achieve tumour negative resection margins is associated with survival benefits and should be the main aim of surgery [23]. Although the complexity and morbidity of the procedure is expectedly reduced by avoiding a complex vascular reconstruction that might compromise mesenteric-portal anastomosis, this should be balanced against the risks of SPH and GI bleeding if limited alternative routes for SV drainage are available. This also requires further consideration when prolonged survival is anticipated in patients with favourable prognostic features.

In patients who had SVL or when SVR is no longer patent, the occurrence or timing of clinically significant SPH or complications can be unpredictable. Several reports have described the results of SVL after surgery for pancreatic cancer; however, the follow-up period was variable and radiologic signs of SPH were not uniformly reported. The safety of SVL and the fact that signs of SPH do not necessarily result in clinically significant symptoms in the majority of cases have been highlighted [2,3,4,22]. Yu et al. reported no GI bleeding over a follow-up period of 19.3 ± 2.4 months in 43 patients who underwent SVL without reconstruction [4].

Conversely, GI bleeding secondary to SPH post pancreaticoduodenectomy has been occasionally reported. Tanaka et al. reported variceal bleeding in 5 out of 87 patients at a median of 17 months post-operatively (range 7–23 months). They also proposed the concept of critical veins (LGV, SRCV, MCV) and highlighted the relationship between the risk of SPH and the number of critical veins preserved in pancreaticoduodenectomy [12]. In another study, bleeding secondary to SPH developed in 1 out of 32 patients who underwent SVL over a median follow-up period of 25 months (interquartile range 16–37 months) [22].

Evidence guiding the decision to either ligate or reconstruct the SV remains inconclusive. However, some useful data might be carefully drawn from meta-analyses and large case series. A systematic review of 8 studies including 337 patients undergoing SVL without reconstruction reported only 14 cases of GI bleeding requiring intervention, with a single mortality. The mean interval from pancreaticoduodenectomy to bleeding was 28 months [16]. Similarly, a large multi-centre study in Japan found that only 9 of 227 patients who had SVL developed variceal bleeding within 3 years postoperatively [17]. These findings suggest that while SPH may develop, clinically significant complications remain relatively uncommon.

### 3.2. Splenic Vein Reconstruction

The primary aim of SVR is to optimize venous drainage and minimize the risk of complications from SPH. Surgical experience in vascular reconstruction is essential to ensure a safe and durable anastomosis. The presence of pre-existing collateral circulation may reduce the need to perform, on many occasions, a complex venous reconstruction. Various techniques with variable levels of complexity have been reported in the literature [6,8,12,19,20,24,25,26,27,28,29,30,31]. The choice of reconstruction technique during pancreatic surgery is influenced by several anatomical factors.

The length and extent of PMJ involvement dictate the suitability of a primary anastomosis or the need for a venous graft. Other factors that need to be considered are the quality of the SV wall and the size-match between the reconstructed veins; the presence of friable or fibrosed vein walls might technically complicate or jeopardize the anastomosis, and a discrepancy in the diameter of the reconstructed veins might result in higher or earlier occlusion rates [8].

Although the direct anastomosis of SV is feasible on occasions [8,24], it is thought to affect Superior Mesenteric Vein (SMV)–Portal Vein (PV) anastomosis by interfering with laminar blood flow, especially if there is an element of tension at the anastomosis site. This can be overcome by utilizing an interposition graft [20,25,26,32] or reconstructing the SV to alternative routes. Vein grafts can be autologous, such as in left renal vein (LRV) [25] or internal jugular vein (IJV) grafts [26], or allogenic cadaveric vein grafts [20]. In cases where surgical expertise allows, the SV can be anastomosed in an end-to-end fashion to the IMV [19,27,28] or end-to-side to the LRV to create a SR shunt [6,8,29,30,31]. More complex reconstructions of SV to the right gonadal vein, the left adrenal vein, or utilizing the IMV as a conduit graft to the LRV or PV have been reported as well [8,12].

Comparative data on different SVR techniques remain limited. Determining the incidence of SPH in relation to the technique and patency of venous reconstruction would be valuable in decision-making. In a study by Ono et al., 30 patients who underwent SVR post pancreaticoduodenectomy were categorized into two groups based on the size of their outflow vessels. End-to-side anastomosis to the PV or LRV (n = 10) had a patency rate of 90%, whereas end-to-end reconstruction to small-calibre veins (n = 20) had a lower patency rate of 45%, with SPH occurring predominantly in the latter group (54%). However, patency rates were evaluated on CT scans at 4–8 months post-surgery, and only one patient developed GI bleeding requiring splenectomy at 40 months [8]. Tanaka et al. reported SV patency in only 17 of 31 (55%) patients who underwent various reconstruction techniques at 6 months post-surgery [12]. The use of SV-LRV anastomosis is advocated by some surgeons due to the conceivably higher patency rates and technical feasibility of reconstruction [6,29,33], though this has not been uniformly reported [31].

Similar to SVL, the outcomes after SVR were not consistent. Ferreira et al. reported no significant short-term changes in the spleen volume or platelet count in 16 patients who underwent SV-IMV reconstruction compared to 11 patients who had SVL with preservation of the IMV-SV junction [19]. In other circumstances, a significant increase in the splenic volume or decrease in the platelet count was noted in SVL versus SVR, but this did not generally result in GI bleeding [7,12,20,24]. Although SVR may lower the risk of SPH, its long-term benefits remain uncertain due to variable patency rates and inconsistent outcomes.

Table 1 shows an overview of the studies investigating the impact of SVL and/or SVR on the incidence of SPH, the development of GI bleeding and the time (if reported) to the onset of bleeding.

## 4. Management of Symptomatic Sinistral Portal Hypertension

Given the low incidence of symptomatic SPH following pancreatic cancer resection, reports of interventions for this condition are infrequent. Intervention for bleeding is usually required on an elective basis, though emergency management has occasionally been reported [38,39]. The modalities for bleeding control can be endoscopic, radiological or surgical. Endoscopic variceal treatment is usually the first diagnostic and therapeutic intervention considered, particularly in the acute setting; however, it does not provide a definitive treatment for SPH [35,40,41]. Splenic artery embolization can effectively treat symptomatic SPH with relatively low morbidity [34,40,42,43,44]. Less invasive radiologic percutaneous embolization techniques have also been reported in specialized centres [43].

Intra-operative splenic artery ligation is associated with a decreased risk of variceal formation in cases of SVL [45,46]. Similarly, surgical intervention in the form of splenectomy remains the most effective treatment for SPH and is curative in most cases [8,35,40]. However, with the advent of less invasive modalities, it is no longer considered the first-line option.

## 5. Discussion

The surgical management of the SV during pancreatic resections with the concurrent resection of the PMJ is debatable. SVL is commonly preferred to avoid a complex venous reconstruction that would involve the use of one or two venous grafts (SMV to PV and/or SV to PV/SMV reconstruction). However, this option carries the risk of SPH development. On the contrary, SVR reduces this risk but may add complexity to the venous reconstruction. Furthermore, the proponents of SVR also report the theoretical advantage of a separate inflow route to the liver in case the SMV-PV reconstruction fails. While SPH is not uncommon after SVL, clinically significant consequences, such as GI bleeding, remain infrequent. Additionally, SVR does not guarantee SPH prevention and is generally avoided due to its technical challenges.

The majority of research has addressed the short-term consequences of ligating or reconstructing the SV, but only some has shown long-term results [6,8,12,29,35]. Studies reporting GI bleeding secondary to SPH have primarily done so in patients who presented with bleeding, rather than as a result of systematic surveillance for SPH. This suggests that many cases of SPH go undetected, either due to an asymptomatic course or because patients succumb to their cancer before developing clinically significant symptoms. The low incidence and relatively long latency until clinically significant SPH develops raise questions about whether SVR is necessary, particularly given the timing for the development of clinically significant SPH in comparison to the median survival of pancreatic cancer patients [47,48,49].

Although SVR should hypothetically eliminate the risk of SPH, several studies have demonstrated radiologic evidence of SPH on post-operative scans with a limited number of GI bleeding cases [8,12,16]. In the majority of studies, the role of collateral venous drainage in mitigating the risk of SPH and clinical decision-making has been highlighted. Ono et al. categorized drainage pathways into varicose and non-varicose routes (through spleno-colic collaterals and SR shunts) and emphasized the importance of preserving the right colic marginal vein in reducing the risk of varices [35]. However, the relative importance of a particular collateral route in protecting against SPH is still debatable, with the IMV, LGV and MCV all representing vital direct or indirect tributaries of the SV.

Currently, no established radiologic or clinical features reliably predict the development of clinically significant SPH. Various surrogate markers, such as a low platelet count and splenic volume changes, have been used inconsistently [5,19,20]; however, these parameters lack specificity and have not been validated for predicting GI bleeding or the need for intervention. Intraoperative SV pressure measurement after clamping, along with the magnitude of pressure change before and after clamping, was shown to predict SPH in one study [50], but this finding has not been corroborated by other studies. Additionally, Tanaka et al. demonstrated that all patients who underwent the ligation of three critical veins (LGV, SRCV, MCV) developed SPH [12]. However, the clinical implications of these findings and their integration into surgical decision-making remain uncertain.

In the authors’ opinion, the preservation of IMV drainage into the SV, when anatomically present, is an important determinant of the need to reconstruct the SV. Retrograde flow through IMV is postulated to be adequate in preventing clinically significant SPH [2,34,51], and SPH developing in such cases may arise due to IMV thrombosis. Of note, the SV-IMV confluence is naturally missing in at least 30% of people [1,52]. Others showed that IMV ligation or preservation was not associated with significantly different splenic volume changes and that, on occasions, it can be sacrificed if other collaterals (MCV, LGV) can be preserved [4,5,35]. Kim et al. also showed that in a multivariate analysis, IMV ligation was not significantly associated with SPH [22]. Thus, the preservation of existing anatomical structures could avoid the complications associated with SPH after SVL and that increase the morbidity of a complex pancreatic resection.

**Figure 1:**
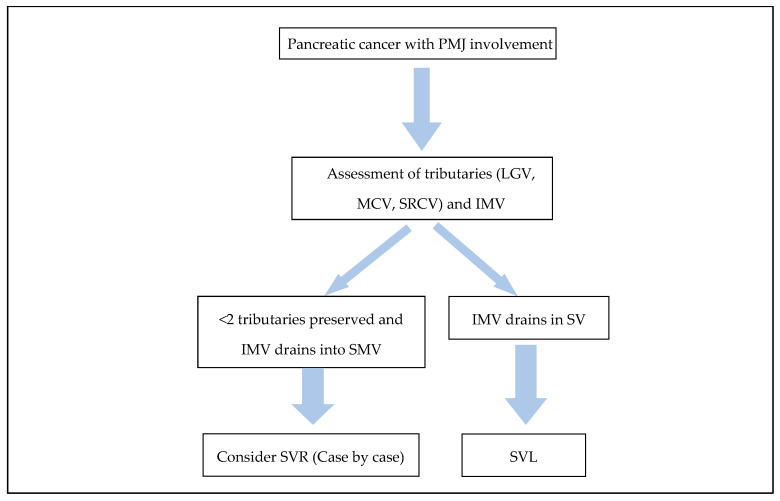
Diagram illustrating SV management during resection of pancreatic cancer involving the PMJ.

With regard to the second argument of SVR providing an alternative route for liver inflow in the case of SMV-PV reconstruction failure, there is no data in the literature. Nonetheless, in order for this to be possible, there are several confounding parameters. Firstly, alternative venous outflow of the bowel should be present in the SV system. This is usually represented by the IMV draining in the SV. In the absence of this though, the failure of the SMV-PV reconstruction will have to be gradual in order to allow for alternative venous outflow pathways to develop. Furthermore, in a complex reconstruction with at least two (PV to SMV and SV to SMV/PV) or usually four venous anastomosis (2-graft reconstruction to avoid tension), if there is failure in one part, then it is likely that the developed thrombus will propagate and occlude the whole reconstruction, thus rendering the SV to PV inflow pathway occluded as well. Therefore, in the absence of clinical evidence, this potential advantage of SVR currently remains theoretical and easily disputed. In Figure 1, we present a flow chart approach to SV management intra-operatively. This diagram is derived from pragmatic clinical experience and the probability of SPH, acknowledging the limited availability of high-quality evidence in this particular domain.

SVL presents a critical surgical decision in pancreatic resectional surgery, particularly in cases where collateral pathways for splenic venous drainage are limited. This may occur due to anatomical variations, the need to divide mesenteric tributaries invaded by the tumour, or the need to facilitate tension-free anastomosis. Currently, there is no definitive evidence in the literature to support routine SVR due to the risks associated with complex venous reconstruction in the rare setting of postoperative clinically significant SPH. However, SVR should be considered in selected cases where the risk of clinically significant SPH may be considered higher, such as expected long-term survival.

## 6. Conclusions

The long-term implications of SV management in pancreatic cancer surgery remain less explored. The development of SPH and its prevention, particularly through precise reconstruction techniques, continues to be a critical area of research to improve patient outcomes. The decision to reconstruct the SV should be highly individualized and considered in the context of the rare occurrence of symptomatic SPH and the availability of different management options. This is also often guided by anatomical factors, disease prognosis and surgical expertise.

## 7. Future Directions

Further research is required to clarify the long-term outcomes of SV management in pancreatic cancer surgery, with a focus on identifying specific predictors of symptomatic SPH that would support SVR. Prospective studies and standardized protocols, even though difficult due to the low incidence of the condition, may help guide individualized decision-making, taking into account anatomical variations, disease prognosis, and surgical expertise.

## Figures and Tables

**Table 1:** Summary of studies evaluating the impact of splenic vein ligation (SVL) and/or reconstruction (SVR) on sinistral portal hypertension (SPH), gastrointestinal (GI) bleeding, and time to bleeding. (N/A: not applicable).

Author et al.	Year	SVL/SVR (No. of Patients)	SPH ^§^ (No. of Patients)	GI Bleeding (No. of Patients)	Time to GI Bleeding
Strasberg et al. [2]	2011	5 SVL	5	0	N/A
Ferreira et al. [19]	2011	11 SVL 16 SVR	Numbers not reported (No significant diff. in plt no. or splenic volume)	0	N/A
Christians et al. [6]	2013	1 SVL 9 SVR ^Ø^	Not reported	1 SVL	2 years
Pilgrim et al. [34]	2014	3 SVL	3	2	1–2 years
Ono et al. [35]	2015	43 SVL	27	3	2–3 years
Hattori et al. [36]	2015	81 SVL	Numbers not reported (low plt/more collaterals in first 6 months)	1	Not reported
Rosado et al. [5]	2017	15 SVL	14	0	N/A
Tanaka et al. [37]	2017	29 SVL	8	0	N/A
Ono et al. [8]	2019	30 SVR	8 ^º^	1	40 months
Tanaka et al. [12]	2019	87 SVL 31 SVR	33 SVL 11 SVR	5 SVL	Median 17 months (7–23 months)
Shiihara et al. [3]	2020	36 SVL	20	0	N/A
Yu et al. [4]	2020	43 SVL	32	0 ^$^	N/A
Addeo et al. [29]	2020	36 SVL 78 SVR	29 SVL 39 SVR	1 SVL	Not reported (patient received treatment at 6 years)
Zhang et al. [20]	2021	13 SVL 24 SVR	4 SVL 0 SVR	0	N/A
Matsuki et al. [24]	2021	11 SVL 3 SVR	Numbers not reported (splenic volume higher in SVL group)	0	N/A
Al-Saeedi et al. [31]	2021	10 SVR	0	0	N/A
Mizuno et al. [17]	2021	227 SVL 24 SVR	84 SVL 9 SVR	9 SVL 1 SVR	Within 3 years (one case within 6 months)
Kim SH et al. [22]	2022	32 SVL ^^^	9	1	Not reported
Wang et al. [7]	2024	23 SVL 43 SVR	8 SVL 3 SVR	2 SVL 0 SVR	Not reported (likely within 6 months)

^§^ Features of SPH reported include splenomegaly, thrombocytopenia, collaterals and/or varices. ^Ø^ Includes 1 patient with neuroendocrine tumour. ^º^ Seven patients had SV reconstructed to small size veins. ^$^ Five patients had positive occult blood. ^^^ Patients who had post operative PV or SMV stricture were excluded.

## Abbreviations

The following abbreviations are used in this manuscript:

SV	Splenic Vein
PMJ	Porto-mesenteric venous Junction
SVL	Splenic Vein Ligation
SVR	Splenic Vein Reconstruction
CT	Computed Tomography
SPH	Sinistral Portal Hypertension
LGV	Left Gastric Vein
IMV	Inferior Mesenteric Vein
MCV	Middle Colic Vein
SRCV	Superior Right Colic Vein
SR	Splenorenal
GI	Gastrointestinal
SMV	Superior Mesenteric Vein
PV	Portal Vein
IJV	Internal Jugular Vein
LRV	Left Renal Vein
N/A	Not Applicable

## References

[1] Sakaguchi T., Suzuki S., Morita Y., Oishi K., Suzuki A., Fukumoto K., Inaba K., Kamiya K., Ota M., Setoguchi T. (2010). Analysis of anatomic variants of mesenteric veins by 3-dimensional portography using multidetector-row computed tomography. Am. J. Surg..

[2] Strasberg S.M., Bhalla S., Sanchez L.A., Linehan D.C. (2011). Pattern of venous collateral development after splenic vein occlusion in an extended Whipple procedure: Comparison with collateral vein pattern in cases of sinistral portal hypertension. J. Gastrointest. Surg..

[3] Shiihara M., Higuchi R., Izumo W., Yazawa T., Uemura S., Furukawa T., Yamamoto M. (2020). Retrospective evaluation of risk factors of postoperative varices after pancreaticoduodenectomy with combined portal vein resection. Pancreatology.

[4] Yu X., Bai X., Li Q., Gao S., Lou J., Que R., Yadav D.K., Zhang Y., Li H., Liang T. (2020). Role of Collateral Venous Circulation in Prevention of Sinistral Portal Hypertension After Superior Mesenteric-Portal Vein Confluence Resection during Pancreaticoduodenectomy: A Single-Center Experience. J. Gastrointest. Surg..

[5] Rosado I.D., Bhalla S., Sanchez L.A., Fields R.C., Hawkins W.G., Strasberg S.M. (2017). Pattern of Venous Collateral Development after Splenic Vein Occlusion in an Extended Whipple Procedure (Whipple at the Splenic Artery) and Long-Term Results. J. Gastrointest. Surg..

[6] Christians K.K., Riggle K., Keim R., Pappas S., Tsai S., Ritch P., Erickson B., Evans D.B. (2013). Distal splenorenal and temporary mesocaval shunting at the time of pancreatectomy for cancer: Initial experience from the Medical College of Wisconsin. Surgery.

[7] Wang J., Lyu S.C., Cui S.P., Huang J.C., Wang H.X., Hu B., He Q., Lang R. (2024). Utilizing bifurcated allogeneic vein grafts: A novel approach for preventing sinistral portal hypertension following Pancreaticoduodenectomy. a 10-year before and after study. Int. J. Surg..

[8] Ono Y., Tanaka M., Matsueda K., Hiratsuka M., Takahashi Y., Mise Y., Inoue Y., Sato T., Ito H., Saiura A. (2019). Techniques for splenic vein reconstruction after pancreaticoduodenectomy with portal vein resection for pancreatic cancer. HPB.

[9] Hadi S., Mehdi S., Arda K., Mustafa F.S. (2014). Splenorenal venous shunt and normal drainage of splenic vein: An unusual case report. Anatomy.

[10] Ai M., Gao D., Lu G., Xu J. (2022). Splenic artery embolization for the treatment of pancreatic portal hypertension complicated by gastric variceal haemorrhage. Gastroenterol. Rev..

[11] Ibukuro K., Ishii R., Fukuda H., Abe S., Tsukiyama T. (2004). Collateral venous pathways in the transverse mesocolon and greater omentum in patients with pancreatic disease. AJR Am. J. Roentgenol..

[12] Tanaka M., Ito H., Ono Y., Matsueda K., Mise Y., Ishizawa T., Inoue Y., Takahashi Y., Hiratsuka M., Unno T. (2019). Impact of portal vein resection with splenic vein reconstruction after pancreatoduodenectomy on sinistral portal hypertension: Who needs reconstruction?. Surgery.

[13] Martin M., Andrea D.G. (2024). Collateral after Splenic Vein Thrombosis. Swiss J. Radiol. Nucl. Med..

[14] Bray F., Laversanne M., Sung H., Ferlay J., Siegel R.L., Soerjomataram I., Jemal A. (2024). Global cancer statistics 2022: GLOBOCAN estimates of incidence and mortality worldwide for 36 cancers in 185 countries. CA Cancer J. Clin..

[15] Park W., Chawla A., O’Reilly E.M. (2021). Pancreatic Cancer: A Review. Jama.

[16] Petrucciani N., Debs T., Rosso E., Addeo P., Antolino L., Magistri P., Gugenheim J., Ben Amor I., Aurello P., D’Angelo F. (2020). Left-sided portal hypertension after pancreatoduodenectomy with resection of the portal/superior mesenteric vein confluence. Results of a systematic review. Surgery.

[17] Mizuno S., Kato H., Yamaue H., Fujii T., Satoi S., Saiura A., Murakami Y., Sho M., Yamamoto M., Isaji S. (2021). Left-sided Portal Hypertension After Pancreaticoduodenectomy With Resection of the Portal Vein/Superior Mesenteric Vein Confluence in Patients With Pancreatic Cancer: A Project Study by the Japanese Society of Hepato-Biliary-Pancreatic Surgery. Ann. Surg..

[18] Kul M., Haliloƒülu N.Ú., Húrsoy N., Erden A. (2018). Sinistral Portal Hypertension: Computed Tomography Imaging Findings and Clinical Appearance-A Descriptive Case Series. Can. Assoc. Radiol. J..

[19] Ferreira N., Oussoultzoglou E., Fuchshuber P., Ntourakis D., Narita M., Rather M., Rosso E., Addeo P., Pessaux P., Jaeck D. (2011). Splenic vein-inferior mesenteric vein anastomosis to lessen left-sided portal hypertension after pancreaticoduodenectomy with concomitant vascular resection. Arch. Surg..

[20] Zhang X., Wu Q., Fan H., He Q., Lang R. (2021). Reconstructing spleno-mesenterico-portal cofluence by bifurcated allogeneic vein in local advanced pancreatic cancer-a feasible method to avoid left-sided portal hypertension. Cancer Med..

[21] Kim S.H., Lee J.M., Choi J.Y., Suh K.S., Yi N.J., Han J.K., Choi B.I. (2008). Changes of portosystemic collaterals and splenic volume on CT after liver transplantation and factors influencing those changes. Am. J. Roentgenol..

[22] Kim S.H., Kim S.S., Hwang H.K., Lee W.J., Kang C.M. (2022). Should the Splenic Vein Be Preserved-Fate of Sinistral Portal Hypertension after Pancreatoduodenectomy with Vascular Re-Section for Pancreatic Cancer. Cancers.

[23] Toomey P., Hernandez J., Morton C., Duce L., Farrior T., Villadolid D., Ross S., Rosemurgy A. (2009). Resection of portovenous structures to obtain microscopically negative margins during pancreaticoduodenectomy for pancreatic adenocarcinoma is worthwhile. Am. Surg..

[24] Matsuki R., Momose H., Kogure M., Suzuki Y., Mori T., Sakamoto Y. (2021). Direct splenic vein reconstruction combined with resection of the portal vein/superior mesenteric vein confluence during pancreaticoduodenectomy. Langenbecks. Arch. Surg..

[25] Miyazaki M., Itoh H., Kaiho T., Ambiru S., Togawa A., Sasada K., Shiobara M., Shimizu Y., Yoshioka S., Yoshitome H. (1995). Portal vein reconstruction at the hepatic hilus using a left renal vein graft. J. Am. Coll. Surg..

[26] Dhakre V.W., Suryawanshi S.S., Shewale V.P., Rathod C., Galande S.T., Sethna K.S. (2022). Successful Use of Direct Splenic Vein Anastomosis to the Interposition Internal Jugular Vein Graft after Extended Pancreatoduodenectomy to Avoid Sinistral Portal Hypertension. Gastrointest. Tumors.

[27] Tamura K., Sumi S., Koike M., Yano S., Nagami H., Nio Y. (1997). A splenic-inferior mesenteric venous anastomosis prevents gastric congestion following pylorus preserving pancreatoduodenectomy with extensive portal vein resection for cancer of the head of the pancreas. Int. Surg..

[28] Addeo P., Nappo G., Felli E., Oncioiu C., Faitot F., Bachellier P. (2016). Management of the splenic vein during a pancreaticoduodenectomy with venous resection for malignancy. Updates Surg..

[29] Addeo P., De Mathelin P., Averous G., Tambou-Nguipi M., Terrone A., Schaaf C., Dufour P., Bachellier P. (2020). The left splenorenal venous shunt decreases clinical signs of sinistral portal hypertension associated with splenic vein ligation during pancreaticoduodenectomy with venous resection. Surgery.

[30] Chavez M.I., Tsai S., Clarke C.N., Aldakkak M., Griffin M.O., Khan A.H., Ritch P.S., Erickson B.A., Evans D.B., Christians K.K. (2019). Distal splenorenal and mesocaval shunting at the time of pancreatectomy. Surgery.

[31] Al-Saeedi M., Frank-Moldzio L., Contin P., Mayer P., Loos M., Schmidt T., Schneider M., Müller-Stich B.P., Berchtold C., Mehrabi A. (2021). Splenorenal shunt for reconstruction of the gastric and splenic venous drainage during pancreatoduodenectomy with resection of the portal venous confluence. Langenbecks Arch. Surg..

[32] Yoshimi F., Asato Y., Tanaka R., Nemoto K., Shioyama Y., Onaya H., Yamada K. (2003). Reconstruction of the portal vein and the splenic vein in pancreaticoduodenectomy for pancreatic cancer. Hepatogastroenterology.

[33] Oba A., Kato T., Inoue Y., Wu Y.H.A., Ono Y., Sato T., Ito H., Saiura A., Takahashi Y. (2021). Extent of venous resection during pancreatectomy-finding the balance of technical possibility and feasibility. J. Gastrointest. Oncol..

[34] Pilgrim C.H., Tsai S., Tolat P., Patel P., Rilling W., Evans D.B., Christians K.K. (2014). Optimal management of the splenic vein at the time of venous resection for pancreatic cancer: Importance of the inferior mesenteric vein. J. Gastrointest. Surg..

[35] Ono Y., Matsueda K., Koga R., Takahashi Y., Arita J., Takahashi M., Inoue Y., Unno T., Saiura A. (2015). Sinistral portal hypertension after pancreaticoduodenectomy with splenic vein ligation. Br. J. Surg..

[36] Hattori M., Fujii T., Yamada S., Inokawa Y., Suenaga M., Takami H., Kanda M., Sugimoto H., Nomoto S., Murotani K. (2015). Significance of the Splenic Vein and Its Branches in Pancreatoduodenectomy with Resection of the Portal Vein System. Dig. Surg..

[37] Tanaka H., Nakao A., Oshima K., Iede K., Oshima Y., Kobayashi H., Kimura Y. (2017). Splenic vein reconstruction is unnecessary in pancreatoduodenectomy combined with resection of the superior mesenteric vein-portal vein confluence according to short-term outcomes. HPB.

[38] Paramythiotis D., Papavramidis T.S., Giavroglou K., Potsi S., Girtovitis F., Michalopoulos A., Papadopoulos V.N., Prousalidis J. (2010). Massive variceal bleeding secondary to splenic vein thrombosis successfully treated with splenic artery embolization: A case report. J. Med. Case Rep..

[39] Gautam A.D., Sanket Agarwal A., Yadav R.R. (2022). Emergent Management of Gastric Variceal Bleed in the Setting of Acute Pancreatitis-Related Sinistral Hypertension With Partial Splenic Embolization: A Series of Two Cases. Cureus.

[40] Liu Q., Song Y., Xu X., Jin Z., Duan W., Zhou N. (2014). Management of bleeding gastric varices in patients with sinistral portal hypertension. Dig. Dis. Sci..

[41] Yang J., Zeng Y., Zhang J.W. (2022). Modified endoscopic ultrasound-guided selective N-butyl-2-cyanoacrylate injections for gastric variceal hemorrhage in left-sided portal hypertension: A case report. World J. Clin. Cases.

[42] Liu J., Meng J., Yang M., Zhou C., Yang C., Huang S., Shi Q., Wang Y., Li T., Chen Y. (2022). Two-step complete splenic artery embolization for the management of symptomatic sinistral portal hypertension. Scand. J. Gastroenterol..

[43] Zhuang Z., Ma J., Zhang Z., Ju S., Gu G., Yang M., Yu J., Yan Z., Zhang W., Luo J. (2024). Endovascular management of sinistral portal hypertension-related variceal hemorrhage: A multicenter retrospective study. Abdom. Radiol..

[44] Patel R.K., Tripathy T., Chandel K., Marri U.K., Giri S., Nayak H.K., Panigrahi M.K., Pattnaik B., Dutta T., Gupta S. (2024). Left-sided portal hypertension: What an interventional radiologist can offer?. Eur. Radiol..

[45] Gyoten K., Mizuno S., Nagata M., Ogura T., Usui M., Isaji S. (2017). Significance of Simultaneous Splenic Artery Resection in Left-Sided Portal Hypertension After Pancreaticoduodenectomy with Combined Portal Vein Resection. World J. Surg..

[46] Yamada D., Takahashi H., Hama N., Toshiyama R., Asukai K., Hasegawa S., Wada H., Sakon M., Ishikawa O. (2021). The clinical impact of splenic artery ligation on the occurrence of digestive varices after pancreaticoduodenectomy with combined portal vein resection: A retrospective study in two institutes. Langenbecks Arch. Surg..

[47] Tang D., Zhang J.Q., Wang D.R. (2011). Long term results of pancreatectomy with portal-superior mesenteric vein resection for pancreatic carcinoma: A systematic review. Hepatogastroenterology.

[48] Ramacciato G., Nigri G., Petrucciani N., Pinna A.D., Ravaioli M., Jovine E., Minni F., Grazi G.L., Chirletti P., Tisone G. (2016). Pancreatectomy with Mesenteric and Portal Vein Resection for Borderline Resectable Pancreatic Cancer: Multicenter Study of 406 Patients. Ann. Surg. Oncol..

[49] Wang J., Lyu S.C., Zhou L., Wang H., Pan F., Jiang T., Lang R., He Q. (2021). Prognostic analysis of pancreatic carcinoma with portal system invasion following curative resection. Gland. Surg..

[50] Ono Y., Takahashi Y., Tanaka M., Matsueda K., Hiratsuka M., Inoue Y., Ito H., Saiura A. (2021). Sinistral Portal Hypertension Prediction During Pancreatoduodenectomy With Splenic Vein Resection. J. Surg. Res..

[51] Misuta K., Shimada H., Miura Y., Kunihiro O., Kubota T., Endo I., Sekido H., Togo S. (2005). The role of splenomesenteric vein anastomosis after division of the splenic vein in pancreatoduodenectomy. J. Gastrointest. Surg..

[52] Nepal P., Mori S., Kita Y., Tanabe K., Baba K., Sasaki K., Kurahara H., Arigami T., Ohtsuka T. (2021). Anatomical study of the inferior mesenteric vein using three-dimensional computed tomography angiography in laparoscopy-assisted surgery for left-sided colorectal cancer. Surg. Today.

